# Origins and Impact of Psychological Traits in Polycystic Ovary Syndrome

**DOI:** 10.3390/medsci7080086

**Published:** 2019-08-05

**Authors:** Elisabet Stener-Victorin, Maria Manti, Romina Fornes, Sanjiv Risal, Haojiang Lu, Anna Benrick

**Affiliations:** 1 Department of Physiology and Pharmacology, Karolinska Institutet, 171 77 Stockholm, Sweden; 2 Centre for Translational Microbiome Research (CTMR), Department of Microbiology, Tumor and Cell biology, Karolinska Institutet, 171 77 Stockholm Sweden; 3 Department of Physiology, Sahlgrenska Academy, University of Gothenburg, 405 30 Gothenburg, Sweden; 4 School of Health and Education, University of Skövde, 541 28 Skövde, Sweden

**Keywords:** PCOS, developmental origin, prenatal androgen exposure, behavior, anxiety, obesity

## Abstract

Women with polycystic ovary syndrome (PCOS) exhibit compromised psychiatric health. Independent of obesity, women with PCOS are more susceptible to have anxiety and depression diagnoses and other neuropsychiatric disorders. During pregnancy women with PCOS display high circulating androgen levels that may cause prenatal androgen exposure affecting the growing fetus and increasing the risk of mood disorders in offspring. Increasing evidence supports a non-genetic, maternal contribution to the development of PCOS and anxiety disorders in the next generation. Prenatal androgenized rodent models reflecting the anxiety-like phenotype of PCOS in the offspring, found evidence for the altered placenta and androgen receptor function in the amygdala, together with changes in the expression of genes associated with emotional regulation and steroid receptors in the amygdala and hippocampus. These findings defined a previously unknown mechanism that may be critical in understanding how maternal androgen excess can increase the risk of developing anxiety disorders in daughters and partly in sons of PCOS mothers. Maternal obesity is another common feature of PCOS causing an unfavorable intrauterine environment which may contribute to psychiatric problems in the offspring. Whether environmental factors such as prenatal androgen exposure and obesity increase the offspring’s susceptibility to develop psychiatric ill-health will be discussed.

## 1. Introduction

Polycystic Ovary Syndrome (PCOS) is a heterogeneous endocrine and metabolic disorder characterized by excessive androgen secretion and abnormal insulin action and affects up to 17% of women worldwide [1]. Women with PCOS are at increased risk of developing symptoms of anxiety and depression and in fact over 60% of women with PCOS are diagnosed with at least one psychiatric disorder [2,3]. Despite the detrimental impact on women’s health, the mechanisms underlying the development of PCOS and anxiety and depression disorders are poorly understood [4]. Although a genetic basis for PCOS has been suggested, the intrauterine milieu might also affect the fetal neurodevelopment and consequently the psychiatric function of a child born to a PCOS mother in a manner that is independent of genetic inheritance or sex. Thus it has been proposed that PCOS and neuropsychiatric disorders originate during fetal development and that this might be, in part, a result of maternal androgen excess causing placental dysfunction and prenatal androgen (PNA) exposure [5]. However, the molecular carriers remain unidentified. This mini-review will discuss the potential impact of PNA exposure and maternal obesity on the development of psychiatric dysfunction and possible mechanisms of action in women with PCOS and in their offspring. The main focus will be on female offspring, but when possible male offspring of PNA mice will also be discussed as it has been shown that sons of women with PCOS are at increased risk for metabolic disturbances [6].

## 2. Anxiety and Depression in Women with Polycystic Ovary Syndrome

The most recent systematic review and meta-analysis shows that women with PCOS have over five times the odds of moderate and severe symptoms of anxiety and over three times the odds of symptoms of depression [2,7]. When controlling for confounding factors such as obesity, the increased risk remains, even if those with symptoms of depression had higher body mass index (BMI) and homeostatic model assessment for insulin resistance (HOMA-IR) [2].

In the recommendations from the international evidence-based guideline for PCOS and in the Androgen Excess and PCOS Society position statement it is stated that health care professionals should be aware of the high prevalence of anxiety and depressive symptoms and that there is strong evidence that these symptoms should be routinely screened in all adolescents and adult women with PCOS at diagnosis [3,8]. 

We performed a new screening of the literature with the search strategy adapted from the Cooney et al. publication [2] and found 31 additional publications related to anxiety and depression in women with PCOS [9,10,11,12,13,14,15,16,17,18,19,20,21,22,23,24,25,26,27,28,29,30,31,32,33,34,35,36,37,38,39,40]. 

## 3. Genetics in Polycystic Ovary Syndrome

Even though PCOS is the most common endocrine and metabolic disorder among women in reproductive age, and is closely linked to anxiety and depressive disorders, the underlying cause of the disorder and related psychiatric ill-health is not known. It is well known that PCOS is a highly familial and heritable disorder and that hyperandrogenism is the most heritable trait in women with PCOS [41]. A recent meta-analysis of genome-wide association studies (GWAS) identified 19 PCOS associated loci and demonstrated that the genetic architecture does not vary across diagnostic criteria used for PCOS [42]. The GWAS meta-analyses also provide the first genetic evidence for a male phenotype for PCOS as well as a causal link to depression. However, the links between PCOS and psychiatric disorders are complicated by BMI and other metabolic variables as these pathways are causal in both PCOS and depression. Furthermore, the proportion of heritability accounted for by the PCOS loci identified so far by GWAS studies is less than 10% [43]. Therefore, other factors increasing susceptibility to psychological traits in this complex disorder remain to be elucidated, including environmental and epigenetic mechanisms. 

## 4. Epigenetics in Polycystic Ovary Syndrome

There are limited human studies that have profiled the epigenome in women with PCOS, although with the new emerging techniques being developed, new data is being generated constantly. One of the first implications of epigenetic involvement in development of PCOS came from studies performed in the PNA exposed rhesus monkey model which found epigenetic changes in adipose tissue of female offspring that could explain the development of a PCOS-like phenotype [44,45]. We and others have later identified specific DNA methylation pathways in ovarian and adipose tissue and in skeletal muscle in women with PCOS that are relevant for development of the disease [44,46,47,48]. However, the latter are cross-sectional observations and do not guide us to whether observed epigenetic changes are due to an altered in utero environment or related to genetic factors. Associations between epigenetic changes and mental complications in PCOS have not yet been explored.

## 5. In Utero Androgen Exposure and Anxiety-Like Behavior in Polycystic Ovary Syndrome Offspring

The observed epigenetic changes in somatic tissues may suggest that PCOS originates during fetal life, where elevated maternal androgens are implicated to play a central role [49]. Women with PCOS retain high circulating androgen levels throughout pregnancy, and these high circulating androgen levels are accompanied by altered placental function with lower aromatase expression potentially leading to higher testosterone exposure of the fetus [50]. The placental function is critical for fetal growth and an altered function is linked with abnormal fetal development, especially of the brain [51]. Moreover, high circulating testosterone levels in women during pregnancy have been shown to alter brain morphology and neural development [52,53], and to be linked to compromised cognitive and neuropsychiatric function in humans [54,55,56,57,58,59]. These clinical observations clearly indicate that elevated maternal androgens may affect the growing fetus and contribute to in utero programming of the developing brain. Of note, a direct indication of PNA exposure of fetuses is demonstrated by longer anogenital distance, a strong marker of in utero androgen exposure, in the newborns of women with PCOS [60]. Further, around 50% of daughters of women with PCOS develop classical symptoms of PCOS, including psychiatric disorders such as anxiety and depression, by adolescence [56,61,62]. 

**Figure 1:**
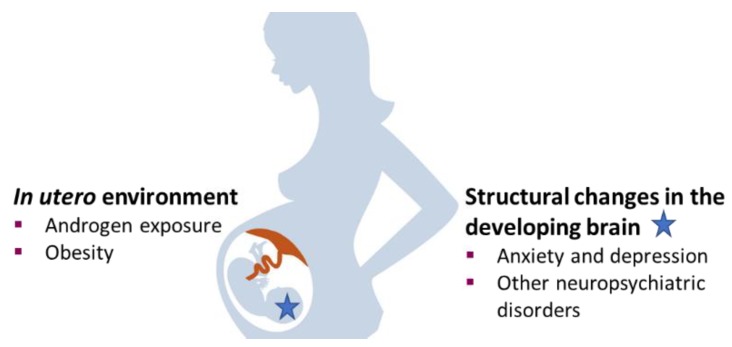
Hypothetical model of prenatal androgen exposure and obesity and the development of psychiatric disorder in the offspring.

Maternal PCOS has been proposed as a model for investigating the role of PNA exposure in the development of neuropsychiatric disorders (Figure 1) [57]. In the recent register-based study it was found that both brothers and sisters of women with PCOS have an increased risk for autism spectrum disorders (ASD), whereas for depressive, anxiety, and schizophrenia spectrum disorders it was found only in the sisters [56]. However, any symptoms observed in offspring of women with PCOS are likely confounded by genetic influences as the mother also present the symptoms. In a recent register-based cohort study they aimed to separate the influence of PNA exposure from familial confounding factors in the offspring development of neuropsychiatric disorders [57]. They show that offspring of women with PCOS have increased risk of being diagnosed with attention-deficit/hyperactivity disorder (ADHD), ASD, and Tourette’s disorder and chronic tic disorders (TD/CTD) compared with unrelated non-PCOS offspring [57]. Girls had a stronger association for ADHD and ASD than boys, but not for TD/CTD. By comparing offspring from PCOS mothers with unrelated offspring from non-PCOS mothers and non-PCOS cousins, thus accounting for genetics and environmental factors shared by cousins, they found evidence for a potential causal factor of PNA exposure, over and above shared familial factors, on the development of male-predominant neuropsychiatric (ADHD and ASD) disorders in female offspring of women with PCOS [57]. However, it should be pointed out that cousins are not necessarily raised in the same environment and frequently are in very different upbringings, locations, and exposures. Despite that, these clinical data support previous observations that PNA exposure may cause anxiety-like behavioral traits, especially in female offspring [63,64].

In a recent study of PNA exposure in rats we found that female offspring, and to a lesser degree male offspring, developed an anxiety-like phenotype [65]. As testosterone is partly converted to estrogen it may act on both androgen and estrogen receptors. Interestingly, simultaneous prenatal administration of flutamide, an androgen receptor blocker, or tamoxifen, an estrogen receptor blocker, reversed the anxiety-like behavioral phenotype due to PNA exposure. This indicates that it is not solely an androgenic effect. To understand the neuroanatomical distribution of sites affected by the PNA treatment we evaluated the gene expression of steroid receptors in these brain regions [65]. We found evidence for disordered androgen receptor function in the amygdala, together with changes in the expression of estrogen receptor-α, serotonergic and gamma-Aminobutyric (GABA)ergic factors in the amygdala and hippocampus of the PNA exposed rats [65]. Further, intra-amygdala testosterone microinjections result in anxiety-like behavior demonstrating that androgens exert an anxiogenic effect in the amygdala. These findings defined a previously unknown mechanism that may be critical in understanding how PNA exposure can increase the risk of developing anxiety disorders in daughters and sons of PCOS mothers. In the same model we also observed placental dysfunction with decreased placenta weight and increased signal transducers and activators of transcription 3 (STAT3) protein signaling which may cause activation of key placental amino acid transporters and negatively affect placental nutrient transport and fetal growth [66], likely due to the PNA exposure [67]. 

In line with these observations, STAT3 was found to be increased in placenta from women with PCOS and in obese women [50]. Importantly, in placenta of women with PCOS, 3β-Hydroxysteroid dehydrogenase (HSD)-1 has been shown to be higher and the aromatase activity (P450) to be lower, which indicate higher androgen production during pregnancy [68]. These findings are supported by elevated circulating androgens in women with PCOS compared with non-PCOS women during the entire pregnancy [50,68].

It is well known that maternal obesity is another common feature of PCOS causing an unfavorable intrauterine environment and is associated with placental dysfunction and altered fetal development [69,70]. It is also suggested that abnormal fetal growth and factors influencing it, can contribute to psychiatric complications in the offspring [71]. 

To explore the impact of maternal obesity in PCOS, we performed another study where we tested the distinct or combined effects of PNA exposure and diet-induced maternal obesity on anxiety-like behavior in female and male offspring [72]. To exclude an estrogenic effect, we used dihydrotestosterone (DHT), a nonaromatizable androgen [72]. We found a sexual dimorphic anxiety-like behavior in the offspring, where PNA exposure induced anxiety-like behavior in female offspring, whereas diet-induced maternal obesity induced anxiety-like behavior in male offspring with no impact on female offspring. Anxiety-like behavior in female offspring was linked to increased expression of adrenergic receptor 1β and corticotrophin releasing hormone receptor 2 in the amygdala. These findings were linked to altered phosphorylation of catechol-*O*-methyltransferase in the PNA exposed placenta, suggesting an altered catecholamine metabolism which might be involved in the development of anxiety-like behavior in female offspring [73]. 

Taken together, emerging evidence suggests that PCOS originates, at least in part, during fetal life and that elevated maternal androgens play a pivotal role and cause placental dysfunction, placing the growing fetus at an increased risk for life-long psychiatric disorders. Whether PNA exposure increases offspring susceptibility to develop anxiety-like behavior in subsequent generations remains to be investigated.
